# ±Genetic structure of the oak wilt vector beetle *Platypus quercivorus*: inferences toward the process of damaged area expansion

**DOI:** 10.1186/1472-6785-10-21

**Published:** 2010-10-15

**Authors:** Etsuko Shoda-Kagaya, Shoichi Saito, Mitsuhiro Okada, Ai Nozaki, Kouichi Nunokawa, Yoshiaki Tsuda

**Affiliations:** 1 Forestry and Forest Products Research Institute, Tsukuba, Japan; 2 Yamagata Prefectural Forest Research and Instruction Center, Sagae, Japan; 3 Nagano Prefecture Forestry Research Center, Shiojiri, Japan; 4 Kyoto Prefecture, Kameoka, Japan; 5 Niigata Prefectural Forest Research Institute, Murakami, Japan; 6 Department of Evolutionary Functional Genomics, Evolutionary Biology Centre, Uppsala University, Uppsala, Sweden

## Abstract

**Background:**

The ambrosia beetle, *Platypus quercivorus*, is the vector of oak wilt, one of the most serious forest diseases in Japan. Population genetics approaches have made great progress toward studying the population dynamics of pests, especially for estimating dispersal. Knowledge of the genetic structuring of the beetle populations should reveal their population history. Using five highly polymorphic microsatellite loci, 605 individuals from 14 sampling sites were assessed to infer the ongoing gene flow among populations as well as the processes of expansion of damaged areas.

**Results:**

Population differentiation (*F*_ST _= 0.047, *G'*_ST _= 0.167) was moderate and two major clusters were detected by several methods, dividing the samples into north-eastern and south-western populations, a similar genetic divergence was reported in host oak trees. Within the north-eastern populations, the subgroups mostly corresponded to differences in the collection period. The genetic characteristics of the population might have changed after 2 years due to the mixing of individuals between populations with enhanced migration related to population outbreaks. Because isolation by distance was detected for whole populations and also within the north-eastern populations, migration was considered to be limited between neighbouring populations, and most populations were suggested to be in genetic equilibrium of genetic drift and gene flow. Recent bottlenecks were found in some populations with no geographical bias; however, they were all from newly emerged oak wilt forests. The emergence of oak wilt should have induced intense fluctuations in the beetle population size.

**Conclusions:**

Because the genetic boundaries coincide, we suggest that the geographical structuring of the beetle was formed by co-evolution with the host species. Our findings indicate the oak wilt expansion process.

## Background

Understanding the processes that drive the spread of plant diseases by vector beetles is critical for developing plans to protect against such damage. Population genetic approaches have made great progress toward studying the population dynamics of pests, especially for estimating dispersal (e.g., [1,2]). Here we report the population genetic structure of an oak forest pest, *Platypus quercivorus *(Murayama; Coleoptera: Platypodidae), and infer the ongoing gene flow among populations.

**Figure 1:**
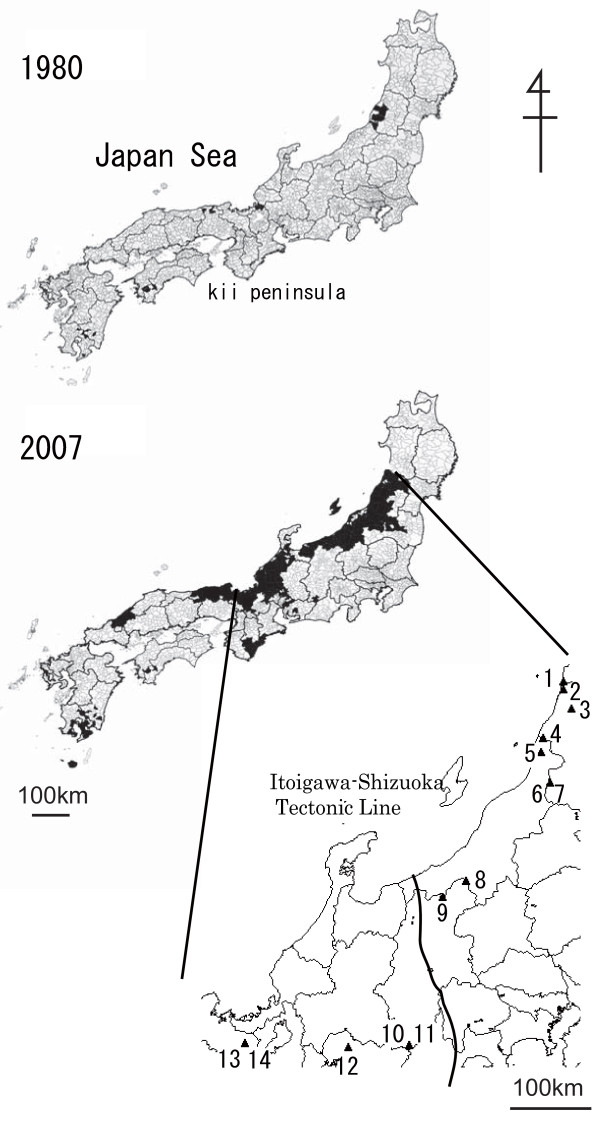
**Spread of oak wilt from 1980 to 2007 and 14 sampling localities of *Platypus quercivorus***. Oak wilt was recorded in black hatched regions from 1980 to 2007 [11].

Japan has lost vast oak tree forests, particularly stands of *Quercus crispula *Blume, due to wilt, at a rate of approximately 2,000 ha/year [3]. This mass mortality of oaks is caused by *Raffaelea quercivora *Kubono et Shin-Ito [4] and its vector, the ambrosia beetle *P. quercivorus *[5,6]. Oak wilt is the first example of a disease caused by an ambrosia fungus carried by an ambrosia beetle that kills vigorous trees [7]. Although the disease was first reported in the 1930s and its emergence has been sporadically reported [8,9], no epidemical symptoms were established before the 1980s [10]. As of 2007, the disease had expanded rapidly, and damage had been reported in 23 of the 46 prefectures in Japan (Figure 1) [11].

The range of the damaged area has been expanding, but where the beetles originated remains unknown. Knowledge of the genetic structuring of the beetle populations should reveal their population history. Such information may also contribute to an understanding of why the oak wilt has been so damaging. Kamata et al. [7] suggested that global climate change has pushed the beetle's distribution northward such that it is now attacking *Q. crispula*, which is a typical species of northern cool-temperate forests in Japan. In addition, oak wilt is less damaging to *Quercus serrata *Thunb. ex Murray, which is more common in southern areas, than to *Q*. *crispula *[12]. Kamata et al. [13] questioned whether fungus, vector and host species are in evolutionary equilibrium to test the invasive species hypothesis. It is possible that *Q*. *crispula *is more susceptible to the disease because of a lack of history of co-evolution between the host and the vector species. Kobayashi and Ueda [14] predicted that changes in the management of oak forests are the main cause of the increase in oak wilt because *P. quercivorus *was widely distributed in Japan before the epidemics. Specifically, they suggested that the beetles use mature trees, whose prevalence has increased in oak forests following the disuse of firewood. Changes in the distribution of the beetles may be traced using population genetics methods, and the cause of the sudden disease expansion may be inferred using molecular ecological studies.

To develop plans for protection against oak wilt, it is necessary to understand the process by which the vector beetles spread the disease. Temporal and spatial patterns of *P. quercivorus *infestations have suggested that the damage spreads from an epicentre to surrounding trees [7]. The majority of disease dispersal is found within the small migration range of the vector; however, damaged areas more than 10 km apart have suddenly emerged [14]. For example, in 2005, Nagano Prefecture reported that after gradual diffusion from the northern region of the prefecture, damaged *Q. serrata *was found 100 km south of the original infestation [15]. It is unclear whether the expansion of the disease is accomplished by colonisation of the vector beetles. There is increasing interest in adopting new technology to estimate dispersal because the movement of organisms from one location to another is integral to the study of evolutionary and ecological dynamics. Genetic markers are valuable tools for analysing the dispersal abilities and mechanisms, and provide an alternative to mark-and-recapture methods (e.g., [16]). Areas of ongoing gene flow are often deduced to describe and interpret patterns of genetic structure. Microsatellite markers in particular exhibit high resolution and are suitable for studying genetic structuring and deducing the ongoing dispersal of pests [17,18]. We used microsatellite markers to map the spatial and temporal population structure of *P. quercivorus *in areas damaged by oak wilt disease. We investigated the dispersal of *P. quercivorus *among populations to thereby gain an understanding of the expansion mechanisms of the disease.

## Methods

### Sampling

In 2005 and 2007, individual beetles were sampled from 14 sites (Table 1, Figure 1). Adult *P. quercivorus *individuals emerge from the trunk of the oak tree and disperse during the summer. We collected adult specimens using an aggregation pheromone [19] and a trap with cross barriers and propylene glycol basins (Sankei). In some newly damaged areas, individuals were collected directly from injured wood or weakened trunks due to the difficulty of trapping flying adults. Samples were collected from damaged areas 0-5 years after the first appearance of oak wilt (Table 1). Site 10 (TRL) represented a population from an area exhibiting newly emergent damage with no reported oak wilt in neighbouring sites. Site 11 (TRT) comprised a population in which no wilting trees were found, and was in close proximity to site 10 (TRL). In Sakae (site 8; SKE), both the flying adults and adults emerging from trunks were collected. All samples were preserved in 99.5% ethanol at -20 or 4°C until DNA extraction.

**Table 1:** Sample sites and sample size of *Platypus quercivorus*

Sampling site and abbreviation	Sampling date	Latitude	Longitude	N	Year of the damage emergence
1) Nikaho, Akita Pref. **NKH**	Jul 9 to Aug 27, 2007	39°07' 34"	139°52' 58"	62	2006
2) Yuza, Yamagata Pref. **YUZ**	Jun 30 to July 13, 2005	39°02' 27"	139°52' 36"	25	2005
3) Sakata, Yamagata Pref. **SAK**	Jul 13, 2005	38°50' 24"	139°59' 18"	29	2004
4) Tsuruoka, Yamagata Pref. **TOK**	Jun 30, 2005	38°31' 48"	139°35' 24"	39	1959, 2004
5) Budou, Asahi, Niigata Pref. **BDU**	Jun 30 to Jul 7, 2005	38°22' 45"	139°33' 06"	33	2003
6) Tamagawa, Oguni, Yamagata Pref. **TMG**	Jul 10 to Aug 30, 2007	38°02' 45"	139°40' 38"	37	2005
7) Ohmiya, Oguni, Yamagata Pref. **OMY**	Jul 26 to Aug 30, 2007	38°04' 29"	139°44' 51"	30	2006
8) Sakae, Nagano Pref. **SKE**	Jul 11 to 25, 2007	36°59' 49"	138°32' 15"	108	2005
	Jul 11 to 17, 2007	36°59' 49"	138°32' 15"*	46	
9) Shinano, Nagano Pref. **SNN**	Jun 30 to July 4, 2005	36°49' 55"	138°13' 17"*	35	2004
10) Tenryu, Nagano Pref. **TRL**	Nov 2, 2005	35°14' 55"	137°47' 38"*	36	2005
11) Tenryu, Nagano Pref. **TRT**	Jul 19 2007	35°14' 48"	137°48' 24"	27	2005
12) Nagoya, Aichi Pref. **NGY**	Oct 25, 2007	35°12' 44"	136°59' 44"*	31	2006
13) Ogadani, Kyoto, Kyoto Pref. **ODN**	Jul 6 to Aug 10, 2005	35°15' 00"	135°39' 36"	47	2000
14) Memedani, Kyoto, Kyoto Pref. **MMD**	Jul 6 to Aug 10, 2005	35°14' 41"	135°38' 23"	20	2000

Total				605	

* Collection from oak timber

N: number of individuals sampled

### DNA extraction and microsatellite genotyping

In total, 20-154 individuals were examined per site (Table 1). Total DNA was extracted using PrepMan reagent (Applied Biosystems) or the Chelex method [20] with some modifications [21]. Five microsatellite loci were examined: Pq3003, Pq3313, Pq3422, Pq3423 and Pq3469 [22]. One primer of each primer set (typically the forward primer) was end-labelled with NED (Pq3422, Pq3423), FAM (Pq3003, Pq3469) or HEX (Pq3313) fluorescent dye. The polymerase chain reaction (PCR) was performed in 8- μl volumes with one-fifth volume of the 5 × PCR buffer Ampdirect containing Mg^2+ ^(Shimadzu Biotech) or one-tenth volume of the 10 × PCR buffer supplied with Ex Taq, 160 μM of each dNTP, 0.4 unit of TaKaRa Ex Taq (TaKaRa), 40 ng of fluorescently labelled primer, 40 ng of the corresponding primer (Applied Biosystems) and genomic DNA. Because the primer pairs Pq3469, Pq3423 and Pq3313 are suitable for multiplex PCR, they were added to one tube with 1/3 concentrates of the mixture described above. Amplification was performed as follows: 94°C for 2 min, followed by 30 cycles of 94°C for 30 s, 53°C for 30 s and 72°C for 45 s. For multiplex loading, 0.5 μl of each PCR product and 0.5 μl of GeneScan 400HD [ROX] Standard (Applied Biosystems) were added to 12 μl of deionised formamide. The products were separated using capillary electrophoresis (ABI PRISM 310; Applied Biosystems) and assigned scores using 310 GeneScan software (Applied Biosystems) and by eye.

### Data analysis

Micro-Checker v. 2.2.3 [23,24] was used to check microsatellite data for scoring errors and null alleles. Fundamental genetic parameters were calculated over all loci using the programme POPGENE v. 1.31 [25]. Observed (*H*_O_) and expected heterozygosities (*H*_E_) were calculated to quantify the genetic diversity of each population. Allelic richness (*A*; [26]) was estimated using a fixed sample size of 20. Putative linkage disequilibrium was assessed between all pairs of loci for each population using GENEPOP [27], and the significance of the *P *value was corrected using the sequential Bonferroni procedure [28]. *F *statistics were studied with FSTAT v. 2.9.3 [29], estimated *F*_IS _and *F*_ST _[30] to test for local inbreeding within populations and differentiation between populations. The significance of deviations from Hardy-Weinberg equilibrium within population was tested by randomisation. The significance of the population differentiation was tested assuming Hardy-Weinberg equilibrium within populations by the permutation test. The genetic diversity and genetic components were compared between trap-collected and trunk-collected samples in Sakae using the indices above. The post hoc test was adjusted using the sequential Bonferroni procedure [28]. Since the absolute values of the population differentiation parameter *F*_ST _depend on the level of genetic variations in the examined material, standardised values of *G*'_ST _[31], which always range from 0 to 1, were also calculated using averaged values of intra-population gene diversity (*H*_S_), total gene diversity (*H*_T_) and the *G*_ST _values calculated by SMOGD [32]. BOTTLENECK v. 1. 2. 02 [33] was used to detect recent bottlenecks under the assumptions of the infinite allele mutation model (IAM), and the Wilcoxon signed rank test was applied. Severe reductions in population size were inferred from comparisons between the expected equilibrium gene diversity (*H*_E_) and the observed numbers of alleles.

An analysis of molecular variance (AMOVA) was performed using the programme ARLEQUIN v. 3.01 [34] to test for a geographical structure effect. An AMOVA was also used to partition the genetic variance among groups, among populations within groups and within populations. Groups were defined as individuals collected in the same year. Upon conducting the AMOVA, *F*_ST _was used to quantify the degree of population differentiation. The significance of any differentiation was tested using a permutation method with 10,000 replications. Multidimensional Scaling (MDS) based on Nei's genetic distances *D*_A _[35] calculated using Populations 1.2.30 [36] was used to explore the relationship between geographical site and genetic differentiation. MDS was performed using the programme SYSTAT v. 9.01 [37]. Multidimensional scaling operates directly on dissimilarities; therefore, no assumptions about statistical distribution were necessary [37]. This avoided the distortions seen in classical scaling methods, which assume a linear relationship between values (e.g., principal co-ordinates analysis PCoA) [38]. The fit of the data in two dimensions was measured by the stress factor. A spatial analysis of molecular variance (SAMOVA, [39]) algorithm was used to define the population configuration. Given the number of groups (*K*), the population configuration with the highest differentiation among groups (*Φ*_CT_) was calculated using a simulated annealing procedure by SAMOVA 1.0 [39]. *K *was set between 2 and 9 with 500 independent simulated annealing processes, and the optimum number of population groups for a set of sample populations was estimated by exploring the behaviour of the index *Φ*_CT _for different values of *K*. Clustering of the populations was assessed by generating a neighbour-joining (NJ) tree based on the *D*_A _genetic distance using Populations with a bootstrapping test.

"Isolation by distance" (IBD) was tested as the correlation between genetic and geographical distance, using all population pairs to estimate the regression of *F*_ST_/(1 - *F*_ST_) on a logarithm of distance for populations, as suggested by Rousset [40]. The relationship between genetic differentiation and geographic distance was assessed by the Mantel test [41], with 9,999 randomisations using GenAlEx 6.2 [42]. This test was done for total populations as well as populations collected in same year (2005 and 2007). The reasoning for the latter analysis is discussed in the Results.

To assess the level of population structure and assignment of an individual's origin, an individual-based clustering was performed using the programme STRUCTURE v. 2.3.1 [43]. *F *statistics and AMOVA tests used the sample location as the unit of comparison, whereas the Bayesian model-based methods of STRUCTURE used the individual as the unit, assigning it to the most likely group (cluster). Each individual genotype was used to estimate the proportion of admixture from several demes in an individual's nuclear genome. Five independent runs for each *K *were used, with burn-ins of 20,000 replicates and run lengths of 10,000 replicates. An allele frequency correlated model (*F *model; [44]) and an admixture model were adopted. STRUCTURE v. 2. 3. 1 is also able to make explicit use of sampling location information to provide accurate inferences [45], and the LOCPRIOR model was used. The likelihood of the assignments was evaluated for *K *varying from 1 to 9, and the reliability of the *K *clusters was tested for these values and variances among the trials; *ΔK *was also used to assess the true *K *number [46]. In the *F *model, all *K *clusters are assumed to be diverged from a common ancestral population at the same time, but the model allows the possibility that the clusters may have experienced different degrees of drift since the divergence event to be considered [47]. In this model, the amount of drift for each cluster from a common ancestral population is described as '*F*', values of which are analogous to traditional *F*_ST _values between clusters and a common ancestral population [44]. Then, *F *for each cluster can be used as an indicator of genetic drift and/or bottleneck in the long term [44,47]. *F *was assessed for each *K *to search for bottlenecks in the long term.

## Results

### Allele polymorphisms and genotypic linkage

We were able to successfully genotype 605 individuals from 14 sampling sites using all five loci. No null alleles or scoring errors were detected by Micro-Checker. The number of alleles varied from 8 (Pq3422) to 16 (Pq3423), with an average of 12.4 alleles per locus. Nine of 150 tests [between Pq3003 and Pq3313 in 1 (NKH); Pq3313 and Pq3469, and Pq3422 and Pq3423 in 9 (SNN); Pq3313 and Pq3423, Pq3313 and Pq3469, and Pq3423 and Pq3469 in 10 (TRL); Pq3313 and Pq3469, Pq3422 and Pq3469, and Pq3422 and Pq3423 in 11 (TRT); *P *< 0.05] showed significant linkage disequilibrium after correcting for multiple tests.

### Genetic diversity and Hardy-Weinberg equilibrium within populations and detection of bottleneck populations

The mean expected heterozygosity (*H*_E_) for all loci varied from 0.594 to 0.741 (Table 2). For most populations, the expected and observed heterozygosities were similar and *F*_IS _values were not significantly different from 0, except for population 1 (NKH; *P *< 0.001). The mean number of alleles (*Ā*) within populations ranged from 4.6 to 7.2. Beetles emerging from trunks and beetles collected using the pheromone trap were similar in their genetic diversity at site 8 (SKE), and no genetic differences were detected between them (*F*_ST _= -0.0018, *P *= 0.59429). Thus, we combined the data for site 8 (SKE) for further analyses.

**Table 2:** Genetic variability estimates of *Platypus quercivorus *samples

Sampling site and abbreviation	*Ā*	***H***_**E**_	***H***_**O**_	*F*is	*P *value in BOTTLENECK
					(Infinite allele mutation model, IAM)
1) **NKH**	5.731	0.694	0.619	0.108***	0.031
2) **YUZ**	4.922	0.599	0.624	-0.042	0.219
3) **SAK**	4.821	0.594	0.614	-0.033	1.000
4) **TOK**	4.813	0.634	0.590	0.071	0.063
5) **BDU**	5.745	0.689	0.667	0.033	0.031
6) **TMG**	6.157	0.727	0.751	-0.034	0.031
7)**OMY**	6.654	0.706	0.687	0.027	0.156
8)**SKE**	5.925	0.735	0.696	0.053	0.031
	5.936	0.741	0.804	-0.086	
9) **SNN**	5.038	0.699	0.794	-0.138	0.813
10)**TRL**	6.311	0.716	0.717	0.000	0.063
11)**TRT**	6.579	0.735	0.696	0.054	0.031
12)**NGY**	4.454	0.687	0.729	-0.063	0.031
13)**ODN**	5.749	0.654	0.626	0.044	1.000
14)**MMD**	5.400	0.631	0.570	0.099	0.063

Abbreviations: *Ā*, mean number of allelic richness [26]; *H*_E_, expected heterozygosity; *H*_O_, observed heterozygosity

Recent genetic bottlenecks were detected within sites 1 (NKH), 5 (BDU), 6 (TMG), 8 (SKE), 11 (TRT), and 12 (NGY; Table 2). Populations showing significant recent bottlenecks were distributed throughout north-eastern and south-western populations; however, they were all from newly emerged oak wilt forests for which the first damage occurred within the 2 years prior to sampling.

### Population differentiations

**Figure 2:**
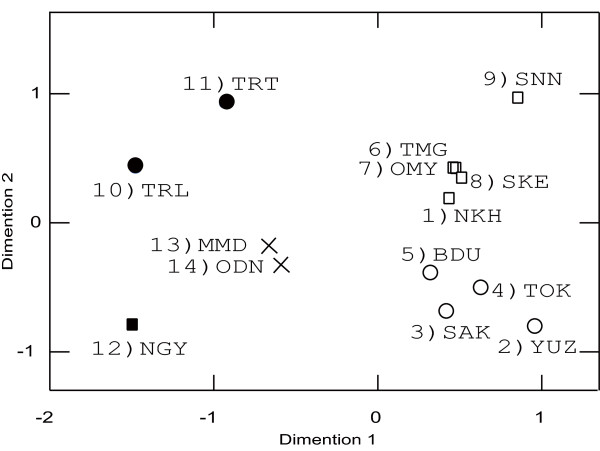
**Multidimensional scaling analysis of *Platypus quercivorus *based on Nei et al.'s distances *D*_A _**[35]. SAMOVA [39] when *K *= 5 is shown using different plot markers which correspond to the NJ tree in Fig 4.

**Figure 3:**
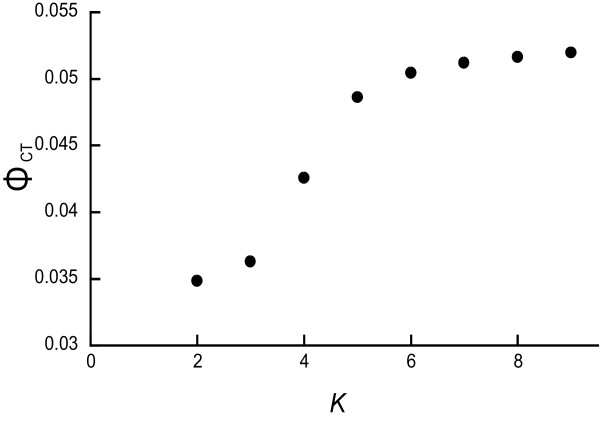
**Plot of the *Φ*_CT _parameter for different values of *K*, the number of population groups generated using SAMOVA **[39].

**Figure 4:**
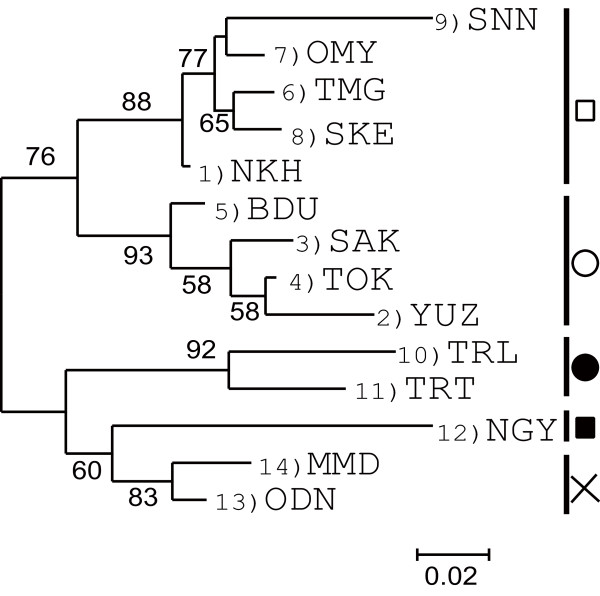
**Neighbour-joining tree of *Platypus quercivorus *generated using Nei et al.'s distances *D*_A _**[35]. Five major clades labelled in symbols correspond to the SAMOVA (*K *= 5) shown in Fig 2. Bootstrap percentages from 1,000 replicates are noted for each branch; only bootstrap values >50% are shown.

Moderate population differentiations were found (*F*_ST _= 0.047 ± 0.002, *G'*_ST _= 0.167 ± 0.001; *P *< 0.001). Pairwise *F*_ST _estimations are shown in Table 3. The AMOVA showed that the variance within populations explained 95.3% of the total variance; 4.7% was expressed among populations and the overall population differentiation was significant (*P *< 0.001). The hierarchical AMOVA indicated that the effect of collection year was marginally significant and the collection site effect was significant (Table 4). The MDS resulted in a stress of the final configuration of 0.084 and explained 96.6% of the variance (Figure 2). The MDS also demonstrated that populations from the south-western region [10 (TRL), 11 (TRT), 12 (NGY), 13 (ODN) and 14 (MMD)] are genetically diverged from populations in the north-eastern region [1 (NKH), 2 (YUZ), 3 (SAK), 4 (TOK), 5 (BDU), 6 (TMG), 7 (OMY), 8 (SKE) and 9 (SNN)]. Two major groups were detected within the north-eastern populations, mostly corresponding to differences in the year of collection (2005 and 2007). The SAMOVA showed that when the number of groups of populations (*K*) increased, the value of *Φ*_CT _increased (Figure 3). From *K *= 2 to 5, *Φ*_CT _increased sharply and then began to level off. Thus, we used *K *= 2-5 for interpreting the grouping patterns. The first level of divergence defined two groups, 12 (NGY) and others. When *K *= 3, population 12 (NGY) and the Tenryu populations, 10 (TRL) and 11 (TRT), are considered to be different groups from the others. In addition, the increase in *K *substantially increased the *Φ*_CT_. Four clusters were revealed as follows: cluster 1 included 1 (NKH), 6 (TMG), 7 (OMY), 8 (SKE) and 9 (SNN); cluster 2 included 2 (YUZ), 3 (SAK), 4 (TOK) and 5 (BDU); cluster 3 included 10 (TRL) and 11 (TRT); and cluster 4 included 10 (NGY), 13 (ODN) and 14 (MMD). Cluster 1 comprises populations distributed in the north-eastern region that were collected in 2007 with the exception of 9 (SNN). Cluster 2 is composed of the 2005 collections, and the distribution of cluster 2 overlaps that of cluster 1. Clusters 3 and 4 are composed of populations from the south-western regions. When *K *= 5, cluster 4 is split in two: one contains10 (NGY) and the other contains 13 (ODN) and 14 (MMD). The clustering of *K *= 4 and *K *= 5 was supported with high bootstrap values in the NJ tree using Nei's *D*_A _(Figure 4). When *K *= 4, two major groups were detected corresponding to clusters 1 and 2 and clusters 3 and 4, supported with 88% and 93% bootstrapping values for clusters 1 and 2, respectively, and by 92% and 60% for clusters 3 and 4. Populations from Kyoto Prefecture [13 (ODN) and 14 (MMD)] exhibited similar genetic components supported by an 83% bootstrapping value in cluster 4.

**Table 3:** Population differentiation estimated *F*_ST _(above the diagonal) and significance for each pairwise comparison (below the diagonal)

	1)	2)	3)	4)	5)	6)	7)	8)	9)	10)	11)	12)	13)	14)
1) **NKH**	-	0.0421	0.0191	0.0299	0.0185	-0.0007	-0.0039	0.0104	0.0301	0.0583	0.0380	0.0638	0.0332	0.0442
2) **YUZ**	*	-	0.0131	-0.0055	0.0184	0.0617	0.0699	0.0630	0.1209	0.1229	0.1114	0.1522	0.0781	0.0927
3) **SAK**	*	NS	-	0.0118	0.0100	0.0410	0.0438	0.0424	0.0913	0.0885	0.0762	0.1064	0.0399	0.0550
4) **TOK**	*	NS	NS	-	0.0083	0.0451	0.0472	0.0511	0.0938	0.0909	0.0766	0.1178	0.0576	0.0626
5) **BDU**	*	NS	NS	NS	-	0.0323	0.0359	0.0279	0.0644	0.0723	0.0473	0.0819	0.0556	0.0594
6) **TMG**	NS	*	*	*	*	-	-0.0035	-0.0001	0.0293	0.0493	0.0357	0.0615	0.0396	0.0490
7) **OMY**	NS	*	*	*	*	NS	-	0.0101	0.0166	0.0617	0.0371	0.0527	0.0314	0.0404
8) **SKE**	*	*	*	*	*	NS	NS	-	0.0352	0.0558	0.0402	0.0669	0.0530	0.0613
9) **SNN**	*	*	*	*	*	*	NS	*	-	0.0957	0.0527	0.0762	0.0763	0.0792
10) **TRL**	*	*	*	*	*	*	*	*	*	-	0.0136	0.0836	0.0809	0.0758
11) **TRT**	*	*	*	*	*	*	*	*	*	NS	-	0.0689	0.0548	0.0493
12) **NGY**	*	*	*	*	*	*	*	*	*	*	*	-	0.0668	0.0538
13) **ODN**	*	*	*	*	*	*	*	*	*	*	*	*	-	-0.0074
14) **MMD**	*	*	*	*	*	*	*	*	*	*	*	*	NS	-

**Table 4:** Hierarchical analysis of molecular variance (AMOVA) of *Platypus quercivorus*

source of variation	**d.f**.	Sum of squares	Variance	% total	Φ statistics	P
Among collection year	1	17.59	0.0108	0.59	*Φ*_CT _= 0.00593	0.0683
Within collection year among sites	12	95.92	0.0787	4.32	*Φ*_SC _= 0.04345	<0.0001
Within sites	1196	2070.79	1.7314	95.09	*Φ*_ST _= 0.00593	<0.0001

Total	1209	2184.30	1.8209			

**Figure 5:**
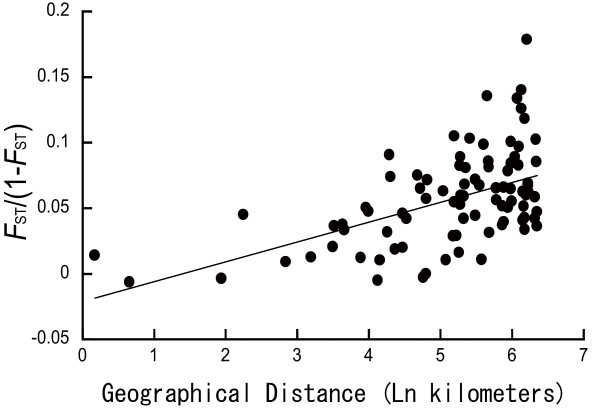
**Relationships between genetic differences [*F*_ST_/(1 - *F*_ST_)] and geographical distance**.

The Mantel test showed that the between-population differentiation increased significantly with geographical distance (*r *= 0.520, *P *< 0.001), as illustrated in Fig 5, suggesting a model of IBD. Given that in north-eastern populations, genetic change was detected between the two collection years (2005 and 2007), the IBD was tested separately for each year. Significant IBD was confirmed for each test (2005, *r *= 0.714, *P *< 0.001; 2007, *r *= 0.495, *P *= 0.001). In addition, the IBD within north-eastern populations in the 2005 collection [2 (YUZ), 3 (SAK), 4 (TOK), 5 (BDU) and 9 (SNN)] was tested to eliminate any effects of differentiation between the north-eastern and south-western populations and the collection year. IBD was also detected in the north-eastern populations for the 2005 collection (*r *= 0.861, *P *= 0.046).

### Individual-based clustering

**Figure 6:**
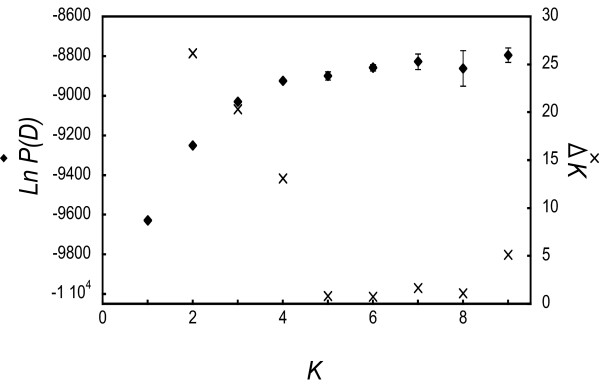
**Estimation of the true number of clusters by STRUCTURE analysis **[43]. Mean likelihood of *K *and the standard deviations of five runs (left axis) and *ΔK *[46] (right axis) for *K *= 1 to 9.

**Figure 7:**
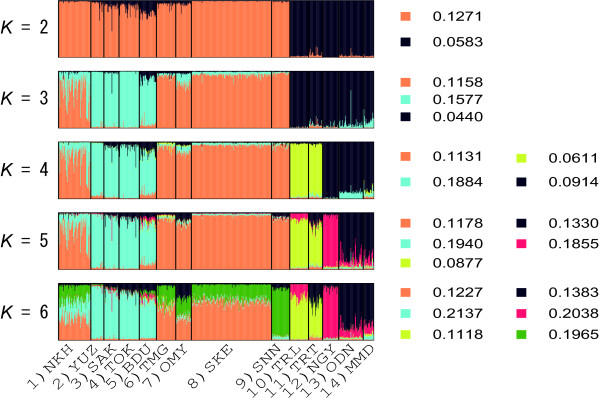
**The proportion of the membership coefficient of 605 individuals in 14 populations for each of the inferred clusters for *K *= 2 to 6 in the STRUCTURE analysis **[43]. Individuals are represented by vertical lines, and the different colours show the probabilities of ancestry from the hypothetical genetic populations partitioned into *K *clusters. The *F *value for each cluster is also shown to the right.

The Bayesian clustering analysis conducted using STRUCTURE showed substantial genetic structuring for *P. quercivorus*. The likelihood of assignment, ln *P*(*D*), increased from *K *= 1 to 7 (Figure 6). It then fluctuated, and the highest value was obtained at *K *= 9. The standard deviation of this value for each iteration increased with the value of *K*, and was very high when *K *> 5. The highest *ΔK *was found at *K *= 2, whereas it decreased to almost 0 when *K *= 5 (Figure 6). Therefore, the consistency of the result was unclear from *K *= 5. Because adding a cluster from *K *= 5-6 did not provide much more information regarding the genetic structuring (Figure 7), assignment results were interpreted for *K *= 2-5. Populations were clearly divided into north-eastern and south-western clusters when *K *= 2, and there appeared to be few migrant individuals. When *K *= 3, the north-eastern cluster was partitioned and most populations exhibited some admixturing, with the exception of 1 (NKH). This partition corresponded to the subgrouping shown in the SAMOVA and NJ analyses. Populations in Tenryu [10 (TNL), 11 (TNT)] were divided from the south-western cluster when *K *= 4. Population 12 (NGY) and populations in the Kyoto prefecture [13 (ODN) and 14 (MMD)] were discriminated when *K *= 5. These partitionings were also consistent with those of the SAMOVA and NJ tree analyses. Higher *F *values were exhibited in the north-eastern clusters when *K *= 2-5 (Figure 7).

## Discussion

### Genetic diversity and population bottleneck

Our data indicate that high genetic diversity exists within populations regardless of the location or collection method. When samples from population 8 (SKE) were compared for their genetic diversity and components, the collection method (trapping flying adults or direct collection from trunks) had little effect on the overall results. Because no null alleles were detected, all of the genotyped data were considered useful for the analysis.

A bottleneck was detected in some populations from recently infested forests. Since there was no damage, these individuals may have originated as a small number of colonisers, which would have entailed a founder effect. If they were not colonisers, they may have been residents, in which case sudden changes in the availability of resources might have resulted in fluctuations in the population size of the beetle in damaged oak forests.

Linkage disequilibrium was also detected in recently infested forest area populations. The lack of linkage disequilibrium in most populations suggests that the loci used may not be in close proximity to one another on the chromosomes. Population bottlenecks and/or admixturing of differentiated populations (mentioned below) might be related to linkage disequilibrium in populations from recently infested areas.

### Oak wilt damage expansion and *P. quercivorus *demographic history

The moderate level of population differentiation (*F*_ST _= 0.047, *G'*_ST _= 0.167) detected in *P. quercivorus *suggests that gene flow among populations is balanced with genetic drift for the entire study area. Genetic analysis using microsatellite markers revealed two main genetic groups of *P. quercivorus *in Japan. These are divided into north-eastern and south-western populations. Using chloroplast DNA, Okaura et al. [48] examined the phylogeography of *Q*. *crispula *and three related species, which are the main hosts of *P. quercivorus*. Quang et al. [49,50] showed nucleotide functional gene variation in *Q*. *crispula *and inferred its history of colonisation into Japan. These studies showed that a genetic boundary for *Q*. *crispula *was found between the north-eastern and south-western populations and coincides with the Itoigawa-Shizuoka Tectonic Line, a major tectonic depression that runs through Nagano Prefecture and separates sites 1 (NKH) through 9 (SNN) from 10 (TRL) through 14 (MMD). Given that the beetles exhibited the same pattern of genetic differentiation between the eastern and western populations, it is possible that they share a population history with *Quercus *species.

A change in the vegetation distribution would likely affect the beetle's genetic structuring in evolutionary timescales. Kamata et al. [7] predicted that *P. quercivorus *expanded its distribution northward and upward in elevation, and that the lack of co-evolution between *Q*. *crispula *and the beetle had an influence on the damage that incurred. They further proposed that a recent warmer climate has altered the distribution of *P. quercivorus *such that they encountered *Q*. *crispula*, which is lacking in defence systems against the disease. Given that the genetic structuring of *P. quercivorus *suggests that the species became established in accordance with the vegetation history on a regional scale, it is difficult to support Kamata et al.'s hypothesis [7] for the main cause of oak wilt expansion in entire regions. Although the genetic structures of the hosts and vectors coincide, the hypothesis that individuals from different origins invaded Japan and caused population structuring divided by the Itoigawa-Shizuoka Tectonic Line by chance cannot be rejected. Further details of the population history of the beetles may be elucidated by additional phylogenetic analysis using individuals from outside Japan.

A rapid expansion of species distribution often entails a lack of geographical structuring [2,51]. The beetle populations that are damaging *Q*. *crispula *in north-eastern areas are not considered exclusively invasive populations because IBD was detected within the north-eastern populations. According to Rousset [40], most populations are thought to be in genetic equilibrium between genetic drift and gene flow. However, we identified a single population that may be composed of migrants. Specifically, the population at site 1 (NKH), which represents the northern limit of oak wilt in 2007, exhibited inbreeding, and an analysis using the STRUCTURE programme predicted that the population was a result of admixturing of two groups. Therefore, the damage at site 1 (NKH) may have been caused by non-local individuals from various source populations.

There was no visible damage near site 10 (TRL), and it was not clear whether the beetles therein that attacked *Q. serrata *were endemic or migrant. Adults were caught by trapping within site 11 (TRT), and there was no oak wilt. Site 11 (TRT) is near to site 10 (TRL). Beetle populations from sites 11 (TRT) and 10 (TRL) showed similar genetic compositions with high genetic diversity. Therefore, they may not represent descendants of long-distance dispersers from severely damaged areas, and may instead be endemic populations. The beetle populations within sites 10 (TRL) and 11 (TRT) must have persisted without a break.

### Population expansion and migration of *P. quercivorus*

The NJ, SAMOVA and clustering by STRUCTURE analyses indicated that within the north-eastern populations, genetic components were substituted between 2005 and 2007. Because IBD was detected among north-eastern populations in 2005, most of the migration should be limited to neighbourhoods. This substitution may have been attained by gradual expansion of population distributions and mixing of population structures prior to the emergence of oak wilt damage. The migration of the beetles among populations might be enhanced by increased oak wilt damage. Therefore, samples collected in 2007 in north-eastern regions may contain individuals from broader areas. Although the beetles may seldom disperse long distances beyond a regional scale, migration within a regional scale may be frequent.

Chapuis et al. [18] examined population outbreak effects using the locust *Locusta migratoria*. They showed that population outbreaks reduce population differentiation by enhancing migration and/or effective population sizes. Thus, an increased *P. quercivorus *population size might alter the population structure in a similar manner to that seen with *L. migratoria*. Our STRUCTURE analysis produced an *F *value that indicated a high value for clustering of individuals in north-eastern populations mostly collected in 2005 when *K *= 3. However, the *F *value decreased in the 2007 collection cluster. This result indicates that the north-eastern population collected in 2005 was influenced by strong genetic drift after divergence from the common ancestral population, and that the effective population size is small. By incorporating independent demes, outbreak populations from 2005 to 2007 might have made the effective population sizes larger.

### Implications for developing protection plans against oak wilt

The genetic structure of the beetles supported Kobayashi and Ueda's prediction [14] that changes in the management of oak forests are the main cause of the increase in oak wilt. Although migrant beetles might occasionally produce new areas of oak wilt damage, our analysis of the genetic structuring of the vector beetle populations indicated that the invasion of the beetle via long-distance dispersal into non-endemic areas or distribution change above regional scale may not be the main cause of oak wilt expansion. Outbreak populations might have enhanced migration on a small scale, and beetles in neighbouring populations may have encouraged such activity. Population outbreaks may also have led to mass attacks on host trees in neighbouring forests, thus establishing new damaged areas. The sequence of activation of the beetle might have promoted disastrous oak wilt.

To prevent the expansion of the disease to undamaged areas, it is important to manage beetle populations to maintain low densities and thus stopping the sequence of activation. Therefore, we need to trap beetles and/or monitor oak wilt damage to provide an indication of the population density of the beetles in continuous oak forests. Once damage is confirmed, the health of the oak forest can be maintained if immediate action is taken before Allee effects (in which the growth rate of sparse populations increases with increasing population density; [52]) become severe. In particular, in mature oak forests composed of plenty of large-diameter trees, vast numbers of beetles can emerge from a few wilted oaks [53]. We must monitor oak forests that have been abandoned for a long time and are full of large trees with caution, so as not to release pests to nearby forests. Further studies of the dispersal dynamics of the beetles, such as frequency and distance of adult flight, are needed to generate alert systems for oak wilt expansion.

## Conclusions

We found genetic differentiation in the ambrosia beetle *P. quercivorus*, which corresponds to the genetic structuring of the host oak trees. Based on a combination of methods of statistical analysis, the genetic structure is presented with a high degree of confidence. The geographical structuring of the beetle was thought to be formed by co-evolution with its host species.

## Authors' contributions

ESK participated in the design of the study, marker selection, genotyping and analyses, and drafted the manuscript. SS, MO, AN and KN collaborated on the study design, sample collection and the interpretation of the results. YT contributed to the improvement of the statistical analyses. All authors read and approved the final manuscript.

## References

[B1] Shoda-Kagaya E Genetic differentiation of the pine wilt disease vector *Monochamus alternatus *over a mountain range - revealed from microsatellite DNA markers Bull Entomol Res 2007 97 167 174 10.1017/S000748530700483X 17411479

[B2] Dalmon A Halkett F Graier M Delatte H Peterschmitt M Genetic structure of the invasive pest *Bemisia tabaci: *evidence of limited but persistent genetic differentiation in glasshouse populations Heredity 2008 100 316 325 10.1038/sj.hdy.6801080 18073781

[B3] Forestry Agency Annual Report on Trends in Forests and Forestry 2006 Tokyo, Japan Forestry Association

[B4] Kubono T Ito S *Raffaelea quercivora *sp. nov. associated with mortality of Japanese oak, and the ambrosia beetle (*Platypus quercivorus*) Mycoscience 2002 43 255 260 10.1007/s102670200037

[B5] Ito S Kubono T Sahashi N Yamada T Associated fungi with the mass mortality of oak trees J Jpn For Soc 1998 80 170 175 (in Japanese with English summary)

[B6] Kinuura H Kobayashi M Death of *Quercus crispula *by inoculation with adult *Platypus quercivorus *(Coleoptera: Platypodidae) Appl Entomol Zool 2006 41 123 128 10.1303/aez.2006.123

[B7] Kamata N Esaki K Kato K Igeta Y Wada K Potential impact of global warming on deciduous oak dieback caused by ambrosia fungus carried by ambrosia beetle in Japan Bull Entomol Res 2002 92 119 126 12020369 10.1079/BER2002158

[B8] Saito K Outbreak of *Crossotarus quercivorus* Forest Pests 1959 87 101 102 (in Japanese)

[B9] Yamazaki S Emergence of Platypodidae damages in Asahi, Niigata Forest Pests 1978 311 28 30 (in Japanese)

[B10] Ito S Yamada T Distribution and spread of mass mortality of oak trees J Jpn For Soc 1998 80 229 232 (in Japanese)

[B11] Takahata Y Kuroda K What is oak wilt? Oak Wilt and Forest Health in Satoyama 2008 Tokyo, Zenrinkyou 25 44 (in Japanese)

[B12] Ichihara Y Masuya H Shoda-Kagaya E Kubono T Relationship between the number of entry holes bored by *Platypus quercivorus *and wilt of oak trees in field conditions Tohoku J For Sci 2009 14 7 11 (in Japanese)

[B13] Kamata N Goto H Komura R Kubo M Mikage M Muramoto K Recent oak wilt in Russia and Korea and prospects in further study for the disease Chubu For Res 2006 54 235 238 (in Japanese)

[B14] Kobayashi M Ueda A Wilt disease of Fagaceae trees caused by *Platypus quercivorus *(Maruyama) (Coleoptera: Platypodidae) and the associated fungus: aim is to clarify the damage factor J Jpn For Soc 2005 87 435 450 (in Japanese with an English summary)

[B15] Nagano Prefecture Occurrence of damage by *Platypus quercivorus* 2005 Nagano (in Japanese)

[B16] Rousset F Colbert J, Danchin E, Dhondt AA, Nichols JD Genetic approaches to the estimation of dispersal rates Dispersal 2001 Oxford, Oxford university press 18 28

[B17] Loxdale HD Lushai G Molecular markers in entomology (review) Bull Entomol Res 1998 88 577 600 10.1017/S0007485300054250

[B18] Chapuis MP Loiseau A Michalakis Y Lecoq M Franc A Estoup A Outbreaks, gene flow and effective population size in the migratory locust, *Locusta migratoria*: a regional-scale comparative survey Mol Ecol 2009 18 792 800 10.1111/j.1365-294X.2008.04072.x 19207256

[B19] Tokoro M Kobayashi M Saito S Kinuura H Nakashima T Shoda-Kagaya E Kashiwagi T Tebayashi S Kim CS Mori K Novel aggregation pheromone, (1S,4R)-p-menth-2-en-1-ol, of the ambrosia beetle, *Platypus quercivorus *(Coleoptera: Platypodidae) Bull FFPRI 2007 6 49 57

[B20] De Lamballerie X Zandott C Vignoli C Bollet C de Micco P A one-step microbial DNA extraction method using "Chelex 100" suitable for gene amplification Res Microbiol 1992 143 785 790 10.1016/0923-2508(92)90107-Y 1298031

[B21] Kawai M Shoda-Kagaya E Maehara T Zhou Z Lian C Iwata R Yamane A Hogetsu T Genetic structure of pine sawyer *Monochamus alternatus *(Coleoptera: Cerambycidae) populations in Northeast Asia: consequences of the spread of pine wilt disease Environ Entomol 2006 35 569 579 10.1603/0046-225X-35.2.569

[B22] Hamaguchi K Kato K Esaki K Kamata N Isolation and characterization of 10 new microsatellite loci in the ambrosia beetle *Platypus quercivorus* J For Res in press

[B23] Brookfield JFY A simple new method for estimating null allele frequency from heterozygote deficiency Mol Ecol 1996 5 453 455 8688964 10.1111/j.1365-294x.1996.tb00336.x

[B24] Van Oosterhout C Hutchinson WF Wills DPM Shipley P MICRO-CHECKER: software for identifying and correcting genotyping errors in microsatellite data Mol Ecol Notes 2004 4 535 538 10.1111/j.1471-8286.2004.00684.x

[B25] Yeh FC Yang RC Boyle TJB Ye ZH Mao JX POPGENE, the user-friendly shareware for population genetic analysis 1997 University of Alberta, Canada, Molecular Biology and Biotechnology Centre http://www.ualberta.ca/~fyeh/

[B26] El Mousadik A Petit RJ High level of genetic differentiation for allelic richness among populations of the argan tree [*Argania spinosa *(L) Skeels] endemic to Morocco Theor Appl Genet 1996 92 832 839 10.1007/BF00221895 24166548

[B27] Raymond M Rousset F Genepop (version 1.2): population-genetics software for exact tests and ecumenicism J Hered 1995 86 248 249

[B28] Rice WR Analyzing tables of statistical tests Evolution 1989 43 223 225 10.2307/2409177 28568501

[B29] Goudet J FSTAT (ver. 1.2): a computer program to calculate *F*-statistics J Hered 1995 86 485 486

[B30] Weir BS Cockerham CC Estimating *F*-statistics for the analysis of population structure Evolution 1984 38 1358 1370 10.2307/2408641 28563791

[B31] Hedrick PW A standardized genetic differentiation measure Evolution 2005 59 1633 1638 16329237

[B32] Crawford NG SMOGD: software for the measurement of genetic diversity Mol Ecol Resources 2010 10 556 557 10.1111/j.1755-0998.2009.02801.x 21565057

[B33] Cornuet JM Luikart G Description and power analysis of two tests for detecting recent population bottlenecks from allele frequency data Genetics 1997 144 2001 2014 10.1093/genetics/144.4.2001 PMC1207747 8978083

[B34] Schneider S Roessli D Excoffier L ARLEQUIN ver. 3.01: a software for population genetics data analysis 2006 Genetics and Biometry Laboratory, University of Geneva

[B35] Nei M Tajima F Tateno Y Accuracy of estimated phylogenic trees from molecular data J Mol Evol 1983 19 153 170 10.1007/BF02300753 6571220

[B36] Langella O Populations 1.2.30: population genetic software (individuals or populations distances, phylogenetic trees) 2007 http://bioinformatics.org/~tryphon/populations/

[B37] SPSS Inc SYSTAT var. 9.01 statistics 1998 Chicago, SPSS Science Marketing Department, SPSS Inc

[B38] Quinn GP Keough MJ Experimental Design and Data Analysis for Biologists 2002 Cambridge: Cambridge University Press

[B39] Dupanloup I Schneider S Excoffier L A simulated annealing approach to define the genetic structure of populations Mol Ecol 2002 11 2571 2581 10.1046/j.1365-294X.2002.01650.x 12453240

[B40] Rousset F Genetic differentiation and estimation of gene flow from F-statistics under isolation by distance Genetics 1997 145 1219 1228 9093870 10.1093/genetics/145.4.1219 PMC1207888

[B41] Mantel N The detection of disease clustering and a generalized regression approach Cancer Res 1967 27 209 220 6018555

[B42] Peakall R Smouse P GENALEX 6: genetic analysis in Excel. Population genetic software for teaching and research Mol Ecol Notes 2006 6 288 295 10.1111/j.1471-8286.2005.01155.x PMC3463245 22820204

[B43] Pritchard JK Stephens M Donnelly P Inference of population structure using multilocus genotype data Genetics 2000 155 945 959 10835412 10.1093/genetics/155.2.945 PMC1461096

[B44] Falush D Stephens M Pritchard JK Inference of population structure: extensions to linked loci and correlated allele frequencies Genetics 2003 164 1567 1587 12930761 10.1093/genetics/164.4.1567 PMC1462648

[B45] Hubisz M Falush D Stephens M Pritchard J Inferring weak population structure with the assistance of sample group information Mol Ecol Resources 2009 9 1322 1332 10.1111/j.1755-0998.2009.02591.x PMC3518025 21564903

[B46] Evanno G Regnaut S Goudet J Detecting the number of clusters of individuals using the software STRUCTURE: a simulation study Mol Ecol 2005 14 2611 2620 10.1111/j.1365-294X.2005.02553.x 15969739

[B47] Tsuda Y Kimura M Kato S Katsuki T Mukai Y Tsumura Y Genetic structure of *Cerasus jamasakura*, a Japanese flowering cherry, revealed by nuclear SSRs: implications for conservation J Plant Res 2009 122 367 375 10.1007/s10265-009-0224-x 19340524

[B48] Okaura T Quang ND Ubukata M Harada H Phylogeographic structure and late Quaternary population history of the Japanese oak *Quercus mongolica *var. *crispula *and related species revealed by chloroplast DNA variation Genes Genet Syst 2007 82 465 477 10.1266/ggs.82.465 18270437

[B49] Quang ND Ikeda S Harada K Nucleotide variation in *Quercus crispula *Blume Heredity 2008 101 166 174 10.1038/hdy.2008.42 18506204

[B50] Quang ND Ikeda S Harada K Patterns of nucleotide diversity at the methionine synthase locus in fragmented and continuous populations of a wind-pollinated tree, *Quercus mongolica *var. *crispula* J Hered 2009 100 762 770 10.1093/jhered/esp036 19531732

[B51] Tsutsui ND Suarez AV Holway DA Case TJ Relationships among native and introduced populations of the Argentine ant (*Linepithema humile*) and the source of introduced populations Mol Ecol 2001 10 2151 2161 10.1046/j.0962-1083.2001.01363.x 11555258

[B52] Drake JM Allee effects and the risk of biological invasion Risk Anal 2004 24 795 802 10.1111/j.0272-4332.2004.00479.x 15357800

[B53] Kobayashi M Shibata M, Togashi K Vector of oak wilt, *Platypus quercivorus* Interesting Life of Insects in the Trees: Invitation to Boring Insect Study 2006 Tokyo, Tokai Univ. Press 89 212 (in Japanese)

